# A Mild Increase in Serum Creatinine after Surgery Is Associated with Increased Mortality

**DOI:** 10.3390/jcm13164905

**Published:** 2024-08-20

**Authors:** Lingyi Xu, Linger Tang, Xizi Zheng, Li Yang

**Affiliations:** 1 Key Laboratory of Renal Disease, Renal Division, Ministry of Health of China, Institute of Nephrology, Peking University First Hospital, Peking University, Beijing 100034, China; lingyi_xu@bjmu.edu.cn (L.X.); 2010301138@bjmu.edu.cn (L.T.); 2 Research Units of Diagnosis and Treatment of Immune-Mediated Kidney Diseases, Key Laboratory of CKD Prevention and Treatment, Ministry of Education of China, Chinese Academy of Medical Sciences, Beijing 100034, China

**Keywords:** acute kidney injury, surgery, MIMIC IV database

## Abstract

**Background**: Acute kidney injury (AKI), a prevalent postoperative complication, predominantly manifests as stage 1, characterized by a mild elevation in serum creatinine (SCr). There is yet to be a consensus regarding the association between stage 1 AKI and adverse outcomes in surgical patients. **Methods:** This retrospective study enrolled adult patients who underwent at least one surgery during hospitalization from the MIMIC IV database. AKI was diagnosed when the KDIGO creatinine criteria were satisfied within 7 days after surgery. Stage 1a AKI was defined as an absolute increase in SCr of 26.5 μmol/L, and stage 1b was defined as a 50% relative increase. Stage 1 AKI was also divided into transient and persistent substages based on whether the AKI recovered within 48 h after onset. The association between stage 1 AKI and its substages and in-hospital mortality was evaluated. **Results:** Among 49,928 patients enrolled, 9755 (19.5%) developed AKI within 7 days after surgery, of which 7659 (78.5%) presented with stage 1 AKI. The median follow-up was 369 (367, 372) days. Stage 1 AKI was significantly associated with in-hospital mortality after adjustment (aHR, 2.73; 95% CI, 2.29, 3.26). Subgroup analyses showed that the risk of stage 1 AKI on in-hospital mortality was attenuated by age ≥ 65 years (*p* for interaction = 0.017) or a baseline eGFR < 60 mL/min per 1.73 m^2^ (*p* for interaction = 0.001). Compared with non-AKI, patients with stage 1b (aHR, 3.06; 95% CI, 2.56, 3.66) and persistent stage 1 (aHR, 2.03; 95% CI, 1.61, 2.57) AKI had an increased risk of in-hospital mortality; however, this risk was not significant in those with stage 1a (aHR, 1.01; 95% CI, 0.68, 1.51) and transient stage 1 (aHR, 1.11; 95% CI, 0.79, 1.57) AKI. **Conclusions:** Stage 1 AKI exhibits an independent correlation with a heightened mortality risk among surgical patients. Consequently, a tailored adaptation of the KDIGO AKI classification may be necessitated for the surgical population, particularly those presenting with decreased baseline kidney function.

## 1. Introduction

Acute kidney injury (AKI), characterized by a sudden decline in kidney function, is a common occurrence among surgical patients, with an incidence ranging from 6% to 26% [1,2,3,4]. The current consensus standard criteria for diagnosing AKI are the Kidney Disease: Improving Global Outcomes (KDIGO) criteria, based on changes in serum creatinine (SCr) and urine output. However, the SCr criteria are more routinely applied in clinical practice due to the challenges associated with accurately quantifying urine output [5]. The clinical manifestations of AKI can range from subclinical to requiring dialysis, and the severity is often classified into three stages (1–3). Stage 1 AKI, defined as an absolute rise in SCr of 26.5 μmol/L or a 1.5- to 2.0-fold relative increase, accounts for as much as 76% of all AKI cases [6,7].

Stage 1 AKI has been robustly demonstrated to correlate with elevated mortality rates in both the short term and long term across the general population. In surgical cohorts, this correlation has not been thoroughly investigated. Most studies focus on a specific surgery type (e.g., cardiac surgery), and results across different studies are inconsistent [7,8,9]. Notably, considerable variations exist even within stage 1 AKI. Patients experiencing a 50% rise from their baseline SCr levels have been observed to exhibit worse prognoses compared to those with an absolute increase of 26.5 µmol/L [10,11]. Divergent outcomes have also been noted in stage 1 AKI with varying durations; specifically, persistent AKI is consistently associated with increased in-hospital mortality [10,12,13] and one-year mortality [12,14,15], whereas transient AKI has not shown such an association [12,16,17,18]. Consequently, we postulate that the relationship between stage 1 AKI and mortality may be influenced by the extent of SCr elevation and the dynamic patterns of AKI reversal.

In the present investigation, we initially assessed the prognostic implications of postoperative stage 1 AKI, designating in-hospital mortality as the primary endpoint and one-year mortality as the secondary endpoint. Subsequently, stage 1 AKI was stratified into distinct substages predicated upon SCr criteria and the temporal duration of AKI. This stratification was conducted to ascertain whether varying subcategories of patients with stage 1 AKI exhibit divergent prognoses.

## 2. Materials and Methods

### 2.1. Study Population

This retrospective cohort study used data from the publicly available Medical Information Mart for Intensive Care IV (MIMIC IV) database, which contains 180,747 de-identified electronic admission records of Beth Israel Deaconess Medical Center in Boston from 2008 to 2019 [19]. All adult patients (aged ≥ 18 years) who underwent at least one surgery during hospitalization were enrolled. Patients were excluded if they (1) had less than two measurements of SCr during hospitalization; (2) were without SCr measurement after surgery; (3) underwent kidney transplantation or nephrectomy; (4) underwent long-term dialysis; (5) had a baseline estimated glomerular filtration rate (eGFR) less than 15 mL/min/1.73 m^2^. Baseline eGFR was calculated according to the Chronic Kidney Disease Epidemiology Collaboration equation (CKD-EPI) using the most recent SCr value within one month before surgery. Data from the first surgery were included for patients who underwent multiple surgeries during a single hospitalization.

### 2.2. Data Collection

The data were extracted using Structured Query Language. Baseline information at the time of surgery included demographic characteristics, chronic comorbidities, preoperative laboratory tests, surgery type, mechanical ventilation, and sequential organ failure assessment (SOFA) scores. Chronic comorbidities were identified according to the admission or discharge diagnosis using the International Classification of Disease (ICD) 10th Revision codes. Preoperative laboratory tests used the most recent values before surgery. Surgery type was classified as cardiac, thoracic, general, genitourinary, orthopedic, neurologic, vascular, and other surgery based on ICD 9th procedures in medicine. Mechanical ventilation was defined as using invasive or non-invasive mechanical ventilation recorded within 24 h of surgery. SOFA score [20] was calculated based on data recorded within 24 h of surgery. Patients were followed up until one-year post-hospital discharge or death. All SCr values during the follow-up period were obtained: baseline SCr was defined as the most recent value within one month before surgery.

### 2.3. Definition and Classification of AKI

AKI was diagnosed when the KDIGO SCr criteria were satisfied within 7 days after surgery and was classified into 3 stages according to the KDIGO AKI classification. Stage 1 AKI was divided into 1a and 1b based on the degree of SCr elevation: stage 1a was defined as an absolute increase in SCr of 26.5 μmol/L, and stage 1b was defined as a 50% relative increase; those who overlapped the two criteria were classified into stage 1b. We also divided stage 1 AKI into transient and persistent categories based on the pattern of reversal: transient stage 1 was defined as when the kidney function recovered to not meet the AKI criteria within 48 h after onset; those who did not meet the criteria of transient AKI were defined as persistent. Duration of AKI was calculated from onset of AKI to discharge.

### 2.4. Clinical Outcomes

The primary outcome was in-hospital mortality, defined as all causes of death until discharge, and the secondary outcome was one-year mortality, defined as all causes of death until one-year post-hospital discharge. The median follow-up was 369 (367, 372) days.

### 2.5. Statistical Analysis

Categorical variables were expressed as frequencies, and a chi-square or Fisher exact test was used to compare groups. Whereas continuous variables were expressed as means ± SDs or medians (interquartile ranges) depending on whether data were normally distributed, non-parametric tests were used for group comparisons. Missing data were not replaced by imputation.

The association between AKI and outcomes was evaluated using the Kaplan–Meier Curve and Cox proportional hazard models. We first tested the crude association in Model 1. In Model 2, we adjusted age, sex, and race. In Model 3, we added baseline eGFR as covariates. In Model 4, we further adjusted comorbidities (hypertension, diabetes, heart failure, coronary heart disease, cerebrovascular disease, chronic pulmonary disease, chronic liver disease, and tumor) and preoperative Hb. In the fully adjusted Model 5, we further adjusted the acute illness status (surgery type, SOFA score, and mechanical ventilation). Hazard ratios (HRs) and 95% confidence intervals (CIs) were reported for each model.

Three sensitivity analyses for the primary outcome were performed. First, the impacts of stage 1a AKI and stage 1b AKI were compared using non-AKI as a reference. Second, the impacts of transient and persistent stage 1 AKI were evaluated; patients who lacked the SCr measurement within 48 h after AKI onset to evaluate the pattern of AKI reversal were excluded. Third, prespecified subgroup analyses were conducted to explore the potential effect modification by age (<65 and ≥65 years), baseline eGFR (<60 and ≥60 mL/min/1.73 m^2^), and cardiac surgery (yes, no).

All analyses were performed using R software (R, version 4.2.1), and statistical testing was conducted at a 2-tailed α level of 0.05.

## 3. Results

### 3.1. Clinical Characteristics

**Figure 1:**
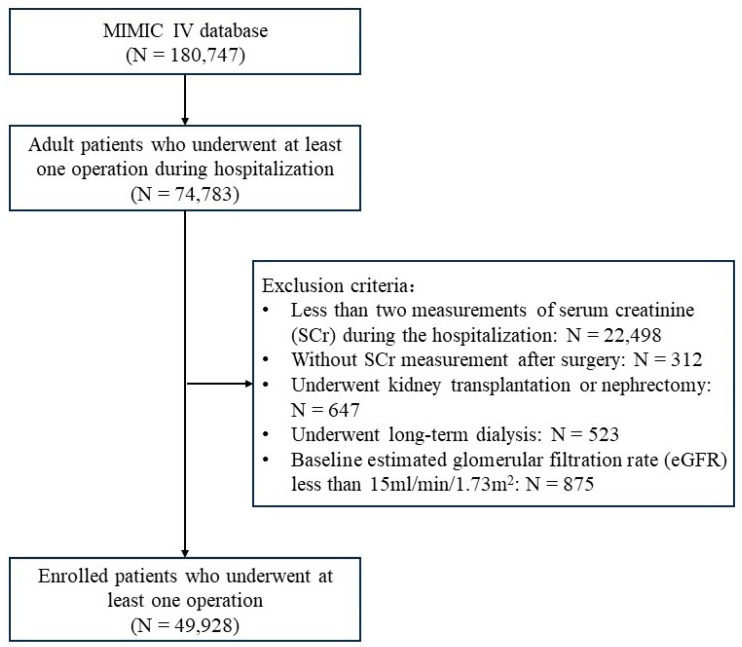
Flowchart of retrospective cohort study using data from the publicly available MIMIC IV database. All adult patients (aged ≥ 18 years) who underwent at least one surgery during hospitalization were enrolled. Patients were excluded if they (1) had less than two measurements of SCr during hospitalization; (2) were without SCr measurement after surgery; (3) underwent kidney transplantation or nephrectomy; (4) underwent long-term dialysis; (5) had a baseline eGFR less than 15 mL/min/1.73 m^2^.

Among 74,783 adult patients who underwent at least one surgery during hospitalization, 49,928 were enrolled (Figure 1). The median age was 65 years old, and 53.8% were males. Hypertension (46.4%), coronary heart disease (30.0%), and diabetes (25.6%) were the most common comorbidities. Orthopedic surgery (20.7%), general surgery (18.5%), vascular surgery (16.7%), cardiac surgery (15.7%), neurologic surgery (12.3%), and thoracic surgery (9.6%) were the most common surgical types. Among 9755 patients who developed AKI within 7 days after surgery, 7659 (78.5%), 925 (9.5%), and 1171 (12.0%) were classified as stage 1, stage 2, and stage 3, respectively. A total of 262 patients (2.7%) underwent renal replacement therapy after AKI onset. The occurrence of AKI was highest in cardiac surgery (41.2%), followed by vascular surgery (21.4%) and thoracic surgery (17.0%). Table 1 summarizes the baseline characteristics of the study population. Compared with non-AKI patients, patients with stage 1 AKI were older, more likely to be male, and had a higher comorbidity burden, including diabetes, heart failure, coronary heart disease, cerebrovascular disease, chronic pulmonary disease, and chronic liver disease. They also presented with lower baseline eGFRs, higher SOFA scores, and a higher proportion of mechanical ventilation.

### 3.2. Association between AKI Stage 1 and In-Hospital Mortality

**Figure 2:**
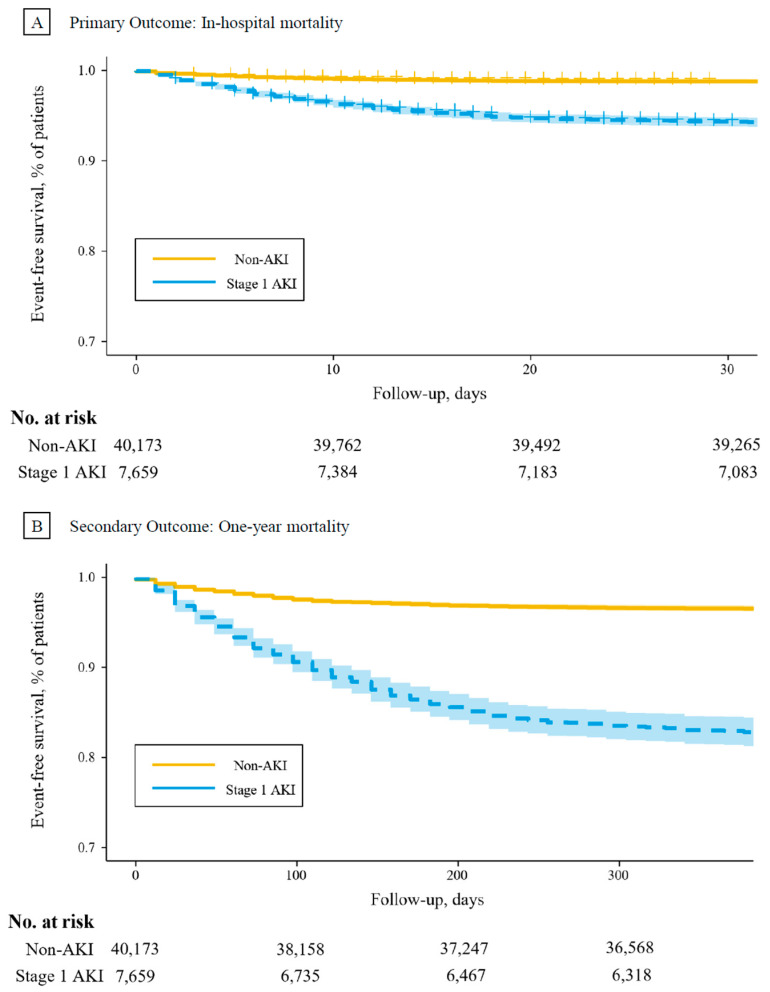
The Kaplan–Meier Curve of mortality. (**A**) Stage 1 AKI was associated with a statistically significant higher risk of in-hospital mortality (HR, 5.29; 95% CI, 4.66, 6.01); (**B**) stage 1 AKI was associated with a statistically significant increased risk of one-year mortality in the unadjusted model (HR, 2.01; 95% CI, 1.89, 2.13).

During a median of 6 (4, 10) days, 1358 (2.7%) patients died during hospitalization. Patients with stage 1 AKI had a significantly higher in-hospital mortality rate than the non-AKI group (6.1% vs. 1.2%, *p* < 0.001). The Kaplan–Meier Curve is shown in Figure 2A. In the unadjusted model, stage 1 AKI was associated with a statistically significant higher risk of in-hospital mortality (HR, 5.29; 95% CI, 4.66, 6.01). This association was attenuated but remained significant after full adjustment (aHR, 2.73; 95% CI, 2.29, 3.26) (Table 2).

Among 7659 patients with stage 1 AKI, 28.5% (2185) were categorized as stage 1a, and 71.5% (5474) were categorized as stage 1b (Table S1). Compared with non-AKI, both stage 1a AKI (HR, 2.87; 95% CI, 2.24, 3.67) and stage 1b AKI (HR, 6.27; 95% CI, 5.49, 7.17) were associated with an increased risk of in-hospital mortality in the unadjusted model. This association was attenuated but remained significant after adjusting for age, sex, race, eGFR, comorbidities, and preoperative Hb. After further adjusting for acute illness status, this association remained significant only in stage 1b AKI (aHR, 3.06; 95% CI, 2.56, 3.66) (Table 3).

**Figure 3:**
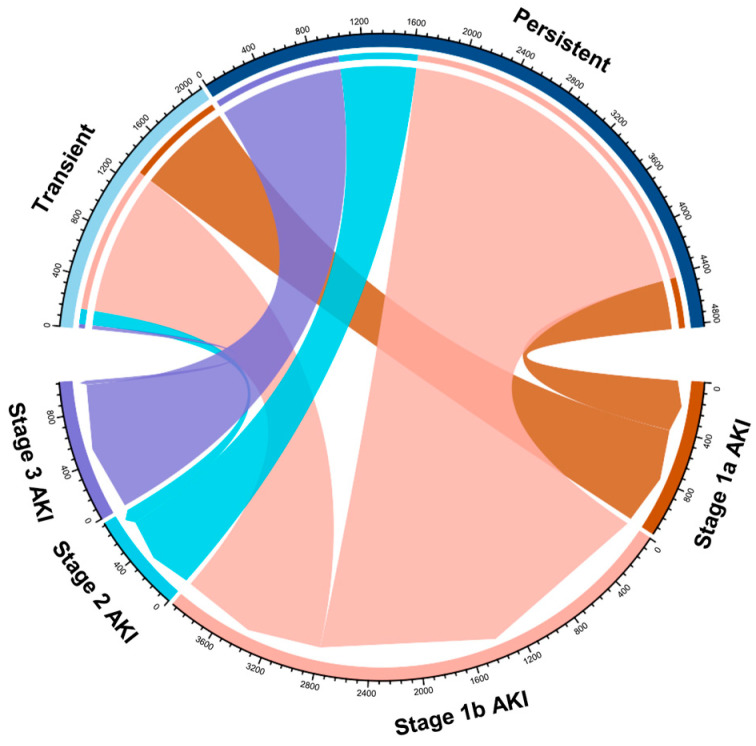
Chord diagram depicting the relationship between AKI severity and duration.

Among 5127 stage 1 AKI patients who had SCr measurements within 48 h after AKI onset, 7.7% (1932) were classified as transient and 66.3% (3195) as persistent. The median durations of AKI were 23 (14, 27) hours for the transient group and 72 (55, 114) hours for the persistent group. The proportions of patients who progressed to stage 2 and stage 3 AKI were 6.0% (116) and 1.7%(33) in the transient group, while they were 19.3% (616) and 31.8% (1015) in the persistent group (Figure 3). Compared with non-AKI, both transient stage 1 AKI (HR, 2.69; 95% CI, 2.06, 3.51) and persistent stage 1 AKI (HR, 5.14; 95% CI, 4.34, 6.08) had significantly increased in-hospital mortality; this association was attenuated but remained significant after adjusting for age, sex, race, eGFR, comorbidities, and preoperative Hb. After further adjusting for acute illness status, only persistent stage 1 AKI was still associated with a significantly increased risk of in-hospital mortality (aHR, 2.03; 95% CI, 1.61, 2.57) (Table 3).

### 3.3. Association between Stage 1 AKI and One-Year Mortality

During a median of 369 (367, 372) days of follow-up, 6366 (12.6%) patients died within one year post-hospital discharge. Patients with stage 1 AKI had a significantly higher one-year mortality rate than the non-AKI group (19.0% vs. 10.0%, *p* < 0.001). The Kaplan–Meier Curve is shown in Figure 2B. Stage 1 AKI was associated with a statistically significant increased risk of one-year mortality in both the unadjusted model (HR, 2.01; 95% CI, 1.89, 2.13) and the fully adjusted model (aHR, 1.25; 95% CI, 1.16, 1.34) (Table 2).

### 3.4. Subgroup Analysis

**Figure 4:**
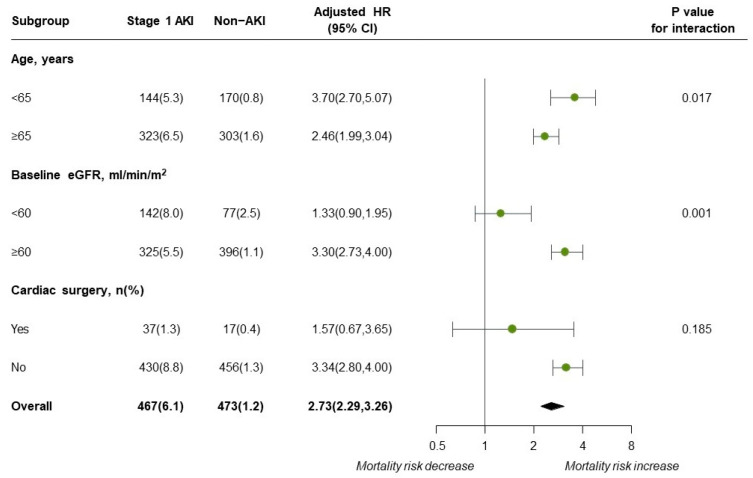
Subgroup analyses of the in-hospital mortality stratified by age, baseline eGFR, cardiac surgery.

As shown in Figure 4, the interaction-term analysis showed an increased risk of in-hospital mortality in association with stage 1 AKI in patients younger than 65 years (*p* for interaction = 0.017) and patients with baseline eGFRs greater than 60 mL/min/1.73 m^2^ (*p* for interaction = 0.001). Cardiac surgery did not modify the effect of stage 1 AKI on in-hospital mortality (*p* for interaction = 0.185).

## 4. Discussion

Postoperative AKI is a common complication associated with increased morbidity and mortality [21,22]. Our study revealed that stage 1 AKI is linked to an elevated risk of in-hospital mortality when considering potential confounders. Subgroup analyses indicated that this risk was mitigated among individuals aged ≥ 65 years, or among those with a baseline eGFR < 60 mL/min per 1.73 m^2^. Within stage 1 AKI, patients with stage 1b and persistent stage 1 AKI had an increased risk of in-hospital mortality; conversely, this risk did not reach statistical significance in patients with stage 1a or transient stage 1 AKI.

Although the detrimental prognostic implications of stage 1 AKI in the broader population are recognized, evidence remains scarce in the context of surgical patients, with consensus yet to be established [7,8,9]. A retrospective cohort analysis of postoperative ICU patients indicated that stage 1 AKI did not exhibit a significant correlation with 28-day mortality in a multivariate analysis (OR, 1.68; 95% CI, 0.85, 3.29) [23]. Parallel outcomes emerged from a prospective cohort examination of subjects undergoing thoracoabdominal aortic aneurysm repair, where the link between stage 1 AKI and 30-day mortality lost significance after adjusting for age, baseline eGFR, and perioperative variables (OR, 2.21; 95% CI, 0.72, 6.79) [24]. Conversely, among patients receiving a gastrectomy for gastric cancer, stage 1 AKI was associated with increased 3-month mortality after the adjustment of age, sex, baseline serum creatinine (SCr), comorbidities, and medications (OR, 3.88; 95% CI, 1.47, 10.25) [25]. A comparable notable relationship was discerned in individuals subjected to cardiopulmonary bypass procedures (OR, 2.432; 95% CI, 1.41, 4.19) [26]. It is pertinent to note that these studies often featured variances in the baseline kidney function. For patients with decreased kidney function, the criterion of an absolute SCr increase of 26.5 μmol/L would ensnare patients exhibiting less pronounced declines in their GFRs relative to those identified using the relative-increase criterion [27]. An extensive database investigation encompassing 1,126,636 American veterans revealed that the risk of mortality tied to AKI was modified by the CKD stages, indicating potential inadequacies of the current AKI definition among patients with pre-existing CKD [28]. This supposition was further corroborated by our data showing that the effects of stage 1 AKI on mortality were attenuated when the baseline eGFR was below 60 mL/min per 1.73 m^2^. In the subgroup analysis, we unexpectedly observed an increased mortality risk of stage 1 AKI in patients under 65 years old and in those with baseline eGFRs ≥ 60. Elderly patients with decreased kidney function were prone to the development of AKI. They were also more likely to have a heavy comorbidity burden and had poor survival. We have reason to speculate that stage 1 AKI may mainly be a marker rather than a mediator of the risk for death in this population. Further research is imperative to ascertain whether a mild increase in SCr is heavily biased by age and baseline kidney function, thereby rendering it unsuitable for the elderly CKD population.

Prior investigations have established that patients categorized within stage 1 AKI who exhibit an increase in SCr of 26.5 μmol/L (stage 1a) manifest clinically substantial and statistically discernible disparities in their mortality rates and hospitalization durations compared to those identified by a 50% relative surge in SCr (stage 1b) [10,11]. Nonetheless, targeted analyses contrasting stage 1a AKI with non-AKI states remain uncommon. Within a sizeable cohort from the general populace, stage 1a AKI was linked to a more than 2.2-fold elevation in the in-hospital mortality risk relative to non-AKI, following adjustment for gender, comorbidities, and admission categorizations [29]. Conversely, a retrospective study reported that stage 1a AKI lacked a significant association with one-year mortality post-surgical intervention when compared with non-AKI cases [30]. The disparate outcomes observed may be attributed to population heterogeneity under the study. Fluctuating SCr levels attributable to hemodynamic shifts during the perioperative phase are frequently observed in surgical patients. Such fluctuations might be readily reversible with fluid management, likely exerting a minimal long-term prognostic impact [31]. It has been postulated that the pattern of AKI reversal reflects the disease severity and impacts postoperative survival [32]. Truche et al. [10] found that patients with transient AKI, defined as kidney recovery occurring within the first 3 days after AKI onset, showed a significantly lower 28-day mortality rate than those with persistent AKI. Choe et al. [33] also reported that within stage 1 AKI, those who recovered within 48 h did not show a significantly increased risk of the composite outcome of new-onset CKD and mortality. Our findings are generally consonant with the extant literature, indicating that individuals with transient stage 1 AKI bear a comparatively diminished mortality risk, suggesting that transient, minor elevations in SCr may not necessarily predict adverse outcomes. Given that stage 1 AKI is common and easily ignored in surgery wards, determining its impact on prognosis is of great importance. 

In this study, the mortality risk of stage 1 AKI, especially stage 1b and persistent stage 1 AKI, was observed in the general surgical population. Risk awareness of AKI should be strengthened, and early recognition and appropriate management is essential. However, several limiting factors still warrant acknowledgment when interpreting our research findings. First, urine output could not be used to assess AKI due to the lack of data on more than half of the study population. Second, we were unable to provide insights into long-term renal prognosis following AKI because of the absence of longitudinal SCr data. Third, variations in the regularity of SCr measurement may potentially render some AKI episodes undetected or misclassified. Fourth, the results of the subgroup analysis require a cautious interpretation due to the limited number of patients in certain subgroups. At last, given the single-center, retrospective nature of our study, which is grounded in the MIMIC database, the applicability of our findings to diverse settings may be constricted.

## 5. Conclusions

In summation, stage 1 AKI is prevalent among surgical patients and exhibits an independent correlation with a heightened mortality risk. Distinct subcategories of stage 1 AKI, delineated by either the magnitude of SCr elevation or the duration of AKI, reflect variances in the severity and clinical outcomes. Consequently, a tailored adaptation of the KDIGO AKI classification may be necessary for the surgical population, particularly those presenting with decreased baseline kidney function.

## Figures and Tables

**Table 1:** Baseline characteristics of enrolled patients who underwent at least one surgery during hospitalization.

Variable	All Patients	Non-AKI	Stage 1 AKI	Stage 2 or 3 AKI	*p* Value *
	N = 49,928	N = 40,173	N = 7659	N = 2096	
Age (y)	65.0 (53.0, 75.0)	64.0 (52.0, 74.0)	70.0 (60.0, 79.0)	66.0 (55.0, 76.0)	<0.001
Male, *n* (%)	26,855 (53.8)	20,844 (51.9)	4774 (62.3)	1237 (59.0)	<0.001
Race, *n* (%)					<0.001
White	35,555 (71.2)	28,760 (71.6)	5375 (70.2)	1420 (67.7)	
Black	4573 (9.2)	3721 (9.3)	648 (8.5)	204 (9.7)	
Others	9800 (19.6)	7692 (19.1)	1636 (21.4)	472 (22.5)	
ICU, *n* (%)	21,463 (43.0)	14, 574 (36.3)	5307 (69.3)	1582 (75.5)	<0.001
Surgery, *n* (%)					<0.001
Cardiac	7855 (15.7)	4617 (11.5)	2767 (36.1)	471 (22.5)	
Thoracic	4818 (9.6)	3998 (10.0)	584 (7.6)	236 (11.3)	
General	9219 (18.5)	7741 (19.3)	1068 (13.9)	410 (19.6)	
Genitourinary	2542 (5.1)	2186 (5.4)	255 (3.3)	101 (4.8)	
Orthopedic	10,359 (20.7)	9151 (22.8)	967 (12.6)	241 (11.5)	
Neurologic	6166 (12.3)	5368 (13.4)	629 (8.2)	169 (8.1)	
Vascular	8316 (16.7)	6539 (16.3)	1333 (17.4)	444 (21.2)	
Other	653 (1.3)	573 (1.4)	56 (0.7)	24 (1.1)	
**Comorbidities, *n* (%)**					
Hypertension	23,188 (46.4)	18,798 (46.8)	3595 (46.9)	795 (37.9)	0.815
Diabetes	12,783 (25.6)	9285 (23.1)	2727 (35.6)	771 (36.8)	<0.001
Heart failure	6086 (12.2)	3881 (9.7)	1724 (22.5)	481 (22.9)	<0.001
Coronary heart disease	14,988 (30.0)	10,604 (26.4)	3596 (47.0)	788 (37.6)	<0.001
Cerebrovascular disease	4957 (9.9)	3787 (9.4)	927 (12.1)	243 (11.6)	<0.001
Chronic pulmonary disease	10,274 (20.6)	7840 (19.5)	1876 (24.5)	558 (26.6)	<0.001
Chronic liver disease	1455 (2.9)	909 (2.3)	342 (4.5)	204 (9.7)	<0.001
Tumor	8543 (17.1)	6939 (17.3)	1164 (15.2)	440 (21.0)	<0.001
**Kidney function**					
Baseline SCr (mg/dL)	0.7 (0.6, 0.9)	0.7 (0.6, 0.9)	0.8 (0.7, 1.1)	0.9 (0.6, 1.3)	<0.001
Baseline eGFR (ml/min/1.73 m^2^)	88.3 (65.7, 102.1)	99.4 (85.9, 110.7)	88.0 (61.9, 101.3)	84.1 (51.7, 104.0)	<0.001
**Preoperative laboratory test**					
WBC (10^9^/L)	7.7 (6.0, 10.0)	7.7 (6.0, 10.0)	7.6 (6.0, 9.8)	8.0 (6.1, 10.8)	<0.001
Hb (g/dL)	12.5 (10.9, 13.8)	12.6 (11.1, 13.9)	12.0 (10.4, 13.5)	11.0 (9.2, 12.9)	<0.001
PLT (10^9^/L)	232.0 (183.0, 294.0)	236.0 (187.0, 297.0)	214.0 (168.0, 273.0)	211.0 (149.0, 289.0)	<0.001
Alb (g/dL)	4.0 (3.5, 4.4)	4.0 (3.6, 4.4)	3.9 (3.5, 4.3)	3.5 (2.9, 4.0)	<0.001
BUN (mg/dL)	17.0 (12.0, 23.0)	16.0 (12.0, 21.0)	21.0 (16.0, 30.0)	20.0 (13.0, 32.0)	<0.001
**Acute illness state, *n* (%)**					
Mechanical ventilation	3411 (6.8)	1766 (4.4)	1057 (13.8)	588 (28.1)	<0.001
SOFA score	0 (0, 1)	0 (0, 1)	1 (0, 3)	2 (0, 4)	<0.001

AKI, acute kidney injury; WBC, white blood cell; Hb, hemoglobin; PLT, platelet count; Alb, albumin; BUN, blood urea nitrogen; SCr, serum creatinine; eGFR, estimated glomerular filtration rate; SOFA, sequential organ failure assessment. * Means comparation was among two groups (non-AKI vs. stage 1 AKI). Missing values: WBC (8988); Hb (8851); PLT (9009); Alb (24,802); BUN (9295).

**Table 2:** Association of stage 1 AKI (versus non-AKI) with mortality in surgical patients.

Outcome	No. of Patients with Events *n* (%)		Hazard Ratio (95% Confidence Interval)				
	Stage 1 AKI	Non-AKI	Model 1	Model 2	Model 3	Model 4	Model 5
**Primary outcome**							
In-hospital mortality	467 (6.1)	473 (1.2)	5.29 (4.66, 6.01)	4.62 (4.05, 5.26)	4.54 (3.97, 5.19)	3.92 (3.32, 4.63)	2.73 (2.29, 3.26)
**Secondary outcome**							
One-year mortality	1453 (19.0)	4035 (10.0)	2.01 (1.89, 2.13)	1.65 (1.55, 1.75)	1.67 (1.57, 1.78)	1.41 (1.31, 1.51)	1.25 (1.16, 1.34)

Model 1: unadjusted; Model 2: adjusted for age, sex, race; Model 3: Model 2 + baseline eGFR; Model 4: Model 3 + comorbidities (hypertension, diabetes, heart failure, coronary heart disease, cerebrovascular disease, chronic pulmonary disease, chronic liver disease, tumor) and preoperative Hb; Model 5: Model 4 + surgery types, SOFA score, and mechanical ventilation. AKI, acute kidney injury; eGFR, estimated glomerular filtration rate.

**Table 3:** The association between different subcategories of stage 1 AKI (stage 1a/1b AKI or transient/persistent stage 1 AKI) and in-hospital mortality.

Sensitivity Analysis	No. of Patients with In-Hospital Mortality (%)		Hazard Ratio (95% Confidence Interval)				
	Alive *n* (%)	Death *n* (%)	Model 1	Model 2	Model 3	Model 4	Model 5
Non-AKI	39,700 (98.8)	473 (1.2)	Reference	Reference	Reference	Reference	Reference
Stage 1a AKI	2112 (96.7)	73 (3.3)	2.87 (2.24, 3.67)	2.44 (1.90, 3.14)	2.30 (1.75, 3.01)	2.17 (1.64, 2.85)	1.01 (0.68, 1.51)
Stage 1b AKI	5080 (92.8)	394 (7.2)	6.27 (5.49, 7.17)	5.42 (4.73, 6.21)	5.35 (4.67, 6.13)	4.89 (4.25, 5.62)	3.06 (2.56, 3.66)
Transient stage 1 AKI	1871 (96.8)	61 (3.2)	2.69 (2.06, 3.51)	2.25 (1.72, 2.95)	2.28 (1.74, 2.99)	2.04 (1.55, 2.68)	1.11 (0.79, 1.57)
Persistent stage 1 AKI	3004 (94.0)	191 (6.0)	5.14 (4.34, 6.08)	4.35 (3.67, 5.16)	4.39 (3.69, 5.23)	3.77 (3.15, 4.51)	2.03 (1.61, 2.57)

Stage 1 AKI was stratified into distinct subcategories based on either the magnitude of SCr elevation (stage 1a or 1b) or the duration of AKI (transient or persistent). As for the transient or persistent subcategories, 2532 stage 1 AKI patients who lacked the SCr value within 48 h after AKI onset were excluded. Model 1: unadjusted; Model 2: adjusted for age, sex, race; Model 3: Model 2 + baseline eGFR; Model 4: Model 3 + comorbidities (hypertension, diabetes, heart failure, coronary heart disease, cerebrovascular disease, chronic pulmonary disease, chronic liver disease, tumor) and preoperative Hb; Model 5: Model 4 + surgery type, SOFA score, and mechanical ventilation. AKI, acute kidney injury; eGFR, estimated glomerular filtration rate; SCr, serum creatinine; SOFA, sequential organ failure assessment.

## Data Availability

The data used in this study are available at the database https://mimic.mit.edu/, accessed on 1 August 2024.

## Supplementary Materials

The following supporting information can be downloaded at https://www.mdpi.com/article/10.3390/jcm13164905/s1, Supplementary Table S1. Baseline characteristics of enrolled patients, categorized into non-AKI, stage 1a AKI, and stage 1b AKI.
