# Supplementary material for: A Mild Increase in Serum Creatinine after Surgery Is Associated with Increased Mortality
Source: J Clin Med. 2024 Aug 20;13(16):4905. doi: 10.3390/jcm13164905 (PMC11355709; doi:10.3390/jcm13164905)
Supplement: Supplementary file 1 [file jcm-13-04905-s001.zip › jcm-3121020-supplementary.pdf]

Supplementary Table S1. Baseline characteristics of enrolled patients, categorized into non-AKI, stage 1a AKI and stage 1b AKI.

Variable	All patients N=49928	Non-AKI N=40173	Stage 1a AKI N= 2185	Stage 1b AKI N = 5474
Age (y)	65.0(53.0,75.0)	64.0(52.0,74.0)	71.0(62.0,79.0)	70.0(59.0,79.0)
Male, n (%)	26855(53.8)	20844(51.9)	1591(72.8)	3183(58.1)
Race, n (%)				
White	35555(71.2)	28760(71.6)	1569(71.8)	3806(69.5)
Black	4573(9.2)	3721(9.3)	188(8.6)	460(8.4)
Others	9800(19.6)	7692(19.1)	428(19.6)	1208(22.1)
**Comorbidities, n (%)**				
Hypertension	23188(46.4)	18798(46.8)	957(43.8)	2638(48.2)
Diabetes	12783(25.6)	9285(23.1)	856(39.2)	1871(34.2)
Heart failure	6086(12.2)	3881(9.7)	511(23.4)	1213(22.2)
Coronary heart disease	14988(30.0)	10604(26.4)	1125(51.5)	2471(45.1)
Cerebrovascular disease	4957(9.9)	3787(9.4)	236(10.8)	691(12.6)
Chronic pulmonary disease	10274(20.6)	7840(19.5)	467(21.4)	1409(25.7)
Chronic liver disease	1455(2.9)	909(2.3)	64(2.9)	278(5.1)
Tumor	8543(17.1)	6939(17.3)	284(13.0)	880(16.1)
**Kidney function**				
Baseline SCr (mg/dL)	0.7(0.6,0.9)	0.7(0.6,0.9)	1.1(0.8,1.4)	0.8(0.6,1.0)
Baseline eGFR (ml/min/1.73m^2)	88.3(65.7,102.1)	99.4(85.9,110.7)	69.2(48.1,91.3)	92.0(71.1,104.5)
**Preoperative laboratory test**				
WBC (10^9/ L)	7.7(6.0,10.0)	7.7(6.0,10.0)	7.5(6.0,9.5)	7.6(6.0,10.0)
Hb (g/dL)	12.5(10.9,13.8)	12.6(11.1,13.9)	12.3(10.7,13.8)	11.9(10.3,13.4)
PLT (10^9/ L)	232.0(183.0,294.0)	236.0(187.0,297.0)	212.0(169.0,261.0)	215.0(168.0,278.0)
Alb (g/dL)	4.0(3.5,4.4)	4.0(3.6,4.4)	4.0(3.7,4.4)	3.9(3.4,4.2)
BUN (mg/dL)	17.0(12.0,23.0)	16.0(12.0,21.0)	23.0(17.0,32.0)	20.0(15.0,29.0)
**Acute illness state, n (%)**				
Cardiac surgery	7855(15.7)	4617(11.5)	759(34.7)	2008(36.7)
Mechanical ventilation	3411(6.8)	1766(4.4)	177(8.1)	880(16.1)

AKI, acute kidney injury; WBC, white blood cell; Hb, hemoglobin; PLT, platelet count; Alb, albumin; BUN, blood urea nitrogen; SCr, serum creatinine; eGFR, estimated glomerular filtration rate; SOFA, sequential organ failure assessment.. Missing value: WBC (8988), Hb (8851), PLT (9009), Alb (24802), BUN (9295).
